# This is a personal journey: a qualitative study on the influencing factors of home-based exercise rehabilitation behavior among stroke survivors

**DOI:** 10.3389/fneur.2026.1736073

**Published:** 2026-01-30

**Authors:** Jianing Shao, Qing Wang, Xiaomin Zhang, Ke Liu, Ling Sha, Huiling Shi, Sunling Cong

**Affiliations:** 1 School of Nursing, Nanjing University of Chinese Medicine, Nanjing, China; 2 School of Drum Tower Clinical Medicine, Nanjing University of Chinese Medicine, Nanjing, China; 3 Department of Discharge Nursing Service Center, Nanjing Drum Tower Hospital, Affiliated Hospital of Medical School, Nanjing University, Nanjing, China; 4 Department of Rehabilitation Medicine, Nanjing Drum Tower Hospital, Affiliated Hospital of Medical School, Nanjing University, Nanjing, China; 5 Department of Neurology, Nanjing Drum Tower Hospital, Affiliated Hospital of Medical School, Nanjing University, Nanjing, China; 6 School of Medicine, Jiangsu University, Zhenjiang, China

**Keywords:** exercise rehabilitation, home, influencing factors, qualitative research, stroke

## Abstract

**Background:**

Adherence with home-based exercise rehabilitation among stroke patients is generally low. Promoting a behavioral shift from passive compliance to active participation in home-based exercise rehabilitation is crucial to address this challenge. However, there is currently a lack of in-depth exploration of the mechanisms underlying this behavioral shift among stroke patients. This study aims to explore the behaviors and influencing factors related to home-based exercise rehabilitation among stroke survivors, thereby providing a reference for the development of personalized home-based exercise rehabilitation programs.

**Methods:**

Seventeen stroke patients who visited the neurology outpatient clinic of a tertiary Grade A hospital in Nanjing, China, from October 2024 to May 2025 were recruited for semi-structured interviews. The Colaizzi seven-step analysis method was applied for thematic analysis and theme extraction.

**Results:**

Analysis of the interview data yielded three core themes and 12 sub-themes. These included: (1) the multidimensional influence of personal characteristics and past experiences (limited body structure and function, driven by family responsibilities, self-decision-making ability, preferences for exercise rehabilitation, and economic pressure burden); (2) the driving and constraints of behavior-related cognition (perceived effects of rehabilitation effects, rehabilitation self-efficacy, adaptability of the rehabilitation environment, and multidimensional information asymmetry); and (3) the moderating effect of behavior-related emotional responses (rehabilitation of emotional experience, family support, and peer support).

**Conclusion:**

Home-based exercise rehabilitation behavior among stroke patients is influenced by multiple complex and interrelated factors. Healthcare professionals should fully consider individual differences among patients, develop tailored exercise rehabilitation programs, and strengthen diversified external support systems to improve adherence to home-based exercise rehabilitation and ultimately enhance long-term rehabilitation outcomes.

## 1 Background

Stroke has become a major global public health issue and is the leading cause of disability among adults, with motor function impairment being the most prominent manifestation (1). Surveys indicate that more than 80% of patients experience varying degrees of motor dysfunction after stroke, and approximately 50% continue to have motor impairments 3 months post-stroke. This results in a significant increase in long-term care and rehabilitation needs, imposing a heavy burden on patients’ families and society (2, 3). The World Stroke Organization recommends exercise rehabilitation as the most effective intervention for promoting motor function recovery in stroke patients and for significantly improving prognosis (4).

However, due to limited rehabilitation resources, more than 50% of stroke patients can only return to home-based rehabilitation following inpatient or outpatient treatment (5). Home-based rehabilitation is a reliable alternative to institutional rehabilitation, not only improving mobility and functional performance in patients with acute or subacute stroke but also offering significant cost-effectiveness advantages (6). Nevertheless, studies consistently report low adherence to home-based exercise rehabilitation among stroke patients. Approximately 80% of patients remain in a passive and sedentary state for prolonged periods during recovery, lacking both the willingness to engage in physical activity and the motivation to persist, which adversely affects rehabilitation outcomes (7, 8). Therefore, facilitating a behavioral shift from passive compliance to active participation in home-based exercise rehabilitation, along with the development of individualized rehabilitation plans and implementation strategies, is critical to addressing the current challenges of home-based exercise rehabilitation.

Accordingly, this study focuses on two primary research objectives: (1) to understand the home-based exercise rehabilitation behaviors of stroke patients, and (2) to identify factors influencing these behaviors and explore corresponding coping strategies.

Motor rehabilitation following stroke is a multifactorial, dynamic, and evolving process. The behavior patterns of patients are deeply rooted in their specific life situations. Although previous research has examined stroke motor rehabilitation, most studies have focused on status surveys (9, 10), needs and experiences (11, 12), or related aspects. Such studies often fail to capture the significance of individual rehabilitation behaviors, the trade-offs involved in decision-making, and the subtle interactions among emotions, environments, and behaviors. Qualitative research methods, which emphasize the perspective of respondents, enable a deeper understanding of subjective experiences and are well-suited to addressing existing gaps in the exploration of behavioral change mechanisms. The Pender’s Health Promotion Model (HPM) focuses on the mechanisms shaping health behaviors and comprises three core components: individual characteristics and experiences (such as past behaviors and personal factors), behavior-specific cognitions and affect (e.g., perceived benefits and barriers, self-efficacy, and situational influences), and behavioral outcomes (13, 14). The model conceptualizes individuals as biopsychosocial beings who interact dynamically with their environments, emphasizing the central role of individual characteristics, experiences, and behavior-specific cognition and emotion in the engagement and maintenance of healthy behaviors (14, 15). To date, HPM has been widely applied in health behavior management for chronic conditions such as hypertension (16), coronary heart disease (17), diabetes (18), and hemodialysis (19), providing a comprehensive theoretical framework for examining factors influencing home-based exercise rehabilitation behaviors. Guided by this model, the present study adopts a qualitative research approach to explore the influencing factors of home-based exercise rehabilitation behaviors among stroke patients, to provide both a theoretical foundation and practical guidance to enhance rehabilitation outcomes.

## 2 Methods

### 2.1 Research design

This study adopted a qualitative research design and conducted semi-structured interviews with stroke patients. The HPM was used as the guiding theoretical framework. Data were analyzed using Colaizzi’s seven-step phenomenological analysis method. In addition, the conduct and reporting of the study followed the Consolidated Criteria for Reporting Qualitative Research checklist (20) to ensure rigor and transparency.

### 2.2 Participants

Objective and maximum variation sampling were used to recruit stroke patients attending follow-up visits at the neurology outpatient department of a tertiary Grade A hospital in Nanjing, China, from October 2024 to May 2025. Inclusion criteria were as follows: (1) meeting the diagnostic criteria outlined in the *Diagnostic Key Points of Major Cerebrovascular Diseases in China (2019)* (21) and having a stroke diagnosis confirmed by cranial CT or MRI; (2) age ≥18 and <80 years; (3) clear consciousness with normal language comprehension and expression abilities; (4) presence of motor dysfunction at discharge, with a modified Rankin Scale (mRS) score ≥2; (5) prior experience with home-based motor rehabilitation; (6) native Chinese speakers. Exclusion criteria included: (1) cognitive impairment or psychiatric disorders; (2) patients with comorbidities such as heart failure, respiratory failure, liver or kidney dysfunction, fractures, or undergoing palliative treatment for malignant tumors; (3) pre-stroke motor dysfunction; (4) patients enrolled in other interview-based studies. To ensure that the sample adequately reflected the diversity of home-based stroke patients, key characteristics—including age, sex, disease duration, functional status, family support structure, and socioeconomic background—were systematically considered during sampling. Participants were recruited by continuously matching newly enrolled individuals with existing participants. According to Bertaux, a minimum of 15 participants is recommended to ensure data adequacy in qualitative research (22). In practice, data collection was discontinued once thematic saturation was achieved, defined as the point at which no new information or themes emerged from additional interviews (23). A total of 17 participants were included in this study.

### 2.3 Collecting tools

Guided by the HPM framework (14), the research team conducted a systematic literature review. It clarified the study objectives before developing a preliminary semi-structured interview outline based on core research questions. Following the initial draft, two rounds of refinement were undertaken. Two stroke patients with experience in home-based exercise rehabilitation were recruited for pre-interviews, and the clarity and relevance of the questions were revised based on their feedback. Subsequently, neurology experts evaluated the interview items for scientific rigor, resulting in a finalized semi-structured interview outline (Box 1).

BOX 1 Semi-structured interview outline.

Open-ended questions tailored to participants.

1. How did you perform exercise rehabilitation at home before? Can you describe it in detail?
2. How do you think your physical function recovery and exercise ability recovery are going? Compared to when you first started home-based rehabilitation, what aspects have improved? What aspects still need improvement?
3. What factors do you think will affect your home-based exercise rehabilitation?
4. What difficulties or obstacles have you encountered during your home-based exercise rehabilitation? How did you solve them?
5. What role did your family members or friends play in your home-based exercise rehabilitation?
6. What kind of help or support do you hope to receive during your home-based exercise rehabilitation?

### 2.4 Data collection

Data were collected through face-to-face semi-structured interviews. Appointments were scheduled in advance, and interviews were conducted after patients had completed their clinical consultations in a quiet and private doctor–patient communication room, with family members permitted to accompany participants if desired. Prior to each interview, the purpose and content of the study were explained, and participants were assured of confidentiality. Interviews commenced only after informed consent was obtained and were recorded. During the interviews, the researcher listened carefully, observed nonverbal behaviors such as body movements and expressions, and conducted an in-depth exploration of relevant questions based on the responses and contextual cues, while avoiding subjective prompts. Field notes were taken to document situational details, emotional expressions, and nonverbal cues. These notes were reviewed alongside the audio transcripts during subsequent analysis to enrich data interpretation and ensure contextual accuracy. A researcher with experience in qualitative research collected all qualitative data.

### 2.5 Data analysis

Within 24 h of each interview, two researchers, both trained in qualitative research, independently transcribed the audio recordings verbatim and annotated contextual information. To protect the privacy of the participants, pseudonyms (e.g., P1 and P2) were assigned. After repeated listening and verification, the transcripts were imported into MAXQDA 2022 for data management and analysis. The HPM was not applied as a predetermined coding framework but was used as an enlightening theoretical framework to guide the analytical process. Interview data were analyzed using Colaizzi’s seven-step analysis method (24): (1) repeated and careful reading of all transcripts; (2) identification and extraction of scientific statements related to the research questions; (3) coding of recurring statements and viewpoints; (4) Clustering of codes into themes; (5) development of detailed thematic descriptions supported by original statements; (6) iterative comparison and refinement of themes to establish final thematic structures; (7) validation of findings by returning them to participants for confirmation. Two researchers independently coded and refined themes, then discussed them jointly. Any discrepancies were resolved through consultation with a third senior researcher.

### 2.6 Quality control

All researchers completed systematic training in qualitative research methods prior to data collection. During interviews, question order and depth were flexibly adjusted to encourage participants to express their ideas. Interviewers maintained neutrality, avoided using guiding sentences, and encouraged the interviewees to fully express their feelings by listening carefully and asking questions and timely clarification. During data analysis, independent coding, regular cross-checking, and thorough discussion were conducted to achieve consensus and ensure coding consistency. All analytical procedures were documented to ensure transparency and traceability. In the later stages of analysis, some respondents were invited to review and verify the extracted themes to confirm alignment with their lived experiences. Throughout the research process, reflexivity was emphasized, and researchers consciously bracketed their preconceptions to minimize subjective bias.

### 2.7 Ethical considerations

This study was reviewed and approved by the Hospital Clinical Research Ethics Committee (approval number: 2024–758-01). All procedures complied with the principles of the Declaration of Helsinki. The privacy and data security of the participants were strictly protected. Written informed consent was obtained from all participants after full disclosure of the study objectives and procedures. Participants were informed of their right to withdraw from the study or seek clarification at any time.

## 3 Results

Seventeen patients were included in the study (Table 1). The average age of the participants was 64 years (range: 55–76 years); 50% were male, and 88% were married. The interviews lasted between 35 and 58 min, with an average duration of 40 min.

**Table 1:** Characteristics of participants.

Participant (P)	Age	Gender	Marital status	Education level	Occupation	Stroke type	Duration of illness (months)	Caregiver
P1	73	Male	Married	Senior middle school	Retired (or resigned)	Ischemic	6	Spouse
P2	59	Male	Married	Junior college	Employee	Ischemic	4	Spouse
P3	68	Female	Married	Middle school	Retired (or resigned)	Ischemic	4	Spouse
P4	59	Male	Married	Junior college	Retired (or resigned)	Ischemic	6	Spouse
P5	60	Male	Married	Senior middle school	Manager	Ischemic	1	Spouse
P6	76	Female	Married	Junior college	Retired (or resigned)	Ischemic	3	Spouse
P7	57	Male	Married	Primary school	Freelance	Ischemic	2	Spouse
P8	57	Male	Married	Middle school	Retired (or resigned)	Ischemic	5	Spouse
P9	55	Male	Married	Primary school	Freelance	Ischemic	2	Spouse
P10	79	Male	Married	Middle school	Retired (or resigned)	Hemorrhagic	1	Spouse
P11	57	Female	Widowed	Illiterate	Freelance	Ischemic	2	Children
P12	68	Female	Divorced	Middle school	Retired (or resigned)	Ischemic	3	None
P13	73	Female	Married	Middle school	Retired (or resigned)	Ischemic	3	Spouse
P14	61	Male	Married	Junior college	Retired (or resigned)	Ischemic	2	Spouse
P15	68	Female	Married	Middle school	Freelance	Ischemic	5	Spouse
P16	60	Female	Married	Middle school	Retired (or resigned)	Hemorrhagic	3	Children
P17	58	Female	Married	Middle school	Retired (or resigned)	Hemorrhagic	2	Spouse

This study ultimately identified three themes and twelve sub-themes (Table 2).

**Table 2:** Overview of themes, subthemes.

Themes	Subthemes
Multidimensional influence of personal characteristics and past experiences	Limited body structure and function
	Driven by family responsibilities
	Self-decision-making ability
	Preferences for exercise rehabilitation
	Economic pressure burden
Driving and constraints of behavior-related cognition	Perceived effects of rehabilitation effects
	Rehabilitation self-efficacy
	Adaptability of the rehabilitation environment
	Multidimensional information asymmetry
Moderating effect of behavior-related emotional responses	Rehabilitation of emotional experience
	Family support
	Peer support

### 3.1 Multidimensional influence of personal characteristics and past experiences

#### 3.1.1 Limited body structure and function

The limitations of body structure and function pose significant challenges for stroke survivors engaging in home-based exercise rehabilitation.

Due to the impact of the disease, most patients experienced symptoms such as reduced muscle strength, abnormal muscle tone, and fatigue. Some patients avoided or refused tedious, repetitive, or high-intensity training tasks, making it difficult to implement and sustain rehabilitation plans effectively.

My arms and legs do not feel like my own. I get tired very easily and have no strength at all… I cannot keep doing those rehabilitation exercises after just a few times. It is really difficult. (P3)

My hand is starting to feel stiff now. Sometimes the muscle tension is so severe that I cannot concentrate on my rehabilitation. I really do not want to continue like this… (P7)

I cannot lift my left leg now, especially for those slightly more complicated and intensive movements. I cannot complete them at all. I know I need to recover, but every time I start training, I feel that my body is just not strong enough. (P12)

#### 3.1.2 Driven by family responsibilities

After experiencing a stroke, some patients faced limb movement dysfunction and expressed a strong desire to recover as soon as possible. They did not want their condition to disrupt their family’s daily routines or become a burden to their loved ones. As a result, these patients were relatively proactive in their rehabilitation efforts and actively sought opportunities to access and utilize rehabilitation services.

My children are already under a lot of pressure working away from home. I want to exercise properly and regain my self-care abilities as soon as possible, so they can feel reassured. That is why I pay close attention to health education programs in the media, hoping to learn more rehabilitation exercises to help me recover faster. (P10)

I saw how the daily rhythm of my family was completely disrupted because of my illness, and it made me feel extremely uncomfortable. I felt I had to get better quickly and could not keep dragging them down. So no matter how hard the training is, I grit my teeth and persist. (P11)

I used to help with all the household chores, but now I have become a burden to everyone. Whenever I think about this, I feel deeply guilty. I took the initiative to ask about different rehabilitation methods and practiced every day. As long as it helps my recovery, I am willing to try. (P16)

#### 3.1.3 Self-decision-making ability

Self-decision-making ability refers to the ability of stroke patients to actively evaluate, select, and implement rehabilitation behaviors based on their understanding of their condition and their awareness of rehabilitation goals. This ability directly influences the autonomy of patients in rehabilitation and the success of long-term self-management in the home environment.

Patients with strong self-decision-making often set goals and self-regulate based on their individual needs, transforming themselves from passive recipients into active leaders of their own recovery.

I set a goal before exercising every day. Every time I achieve it, I encourage myself. If I do not achieve it, I look for the problem and make adjustments. This way, I feel more confident about my recovery.” (P6)

After recovering for some time, I realized that I feel better in the morning and get especially tired in the afternoon. So I adjusted my training schedule to practice more in the morning and less in the afternoon, which helps me avoid excessive fatigue. (P10)

In contrast, patients with low self-decision-making ability tended to rely passively on external guidance, lacked confidence, and encountered systematic difficulties during rehabilitation.

The doctor told me to practice raising my arms, but did not specify how high to raise them or how often to change the movement. I tried practicing on my own, but my shoulders hurt so badly that I cannot move them now. (P5)

I have no confidence in my body and do not dare make decisions on my own. My family bought elastic bands for me before and said they could help strengthen my arms, but when I picked them up, I felt intimidated—unsure how much force to use or how many repetitions to do. (P14)

#### 3.1.4 Preferences for exercise rehabilitation

The preferences of stroke patients for exercise rehabilitation are primarily reflected in their priorities and behavioral tendencies when selecting exercise methods, including exercise type, intensity, setting, assistive tools, and forms of supervision. At its core, decision-making autonomy and behavioral maintenance in home-based exercise rehabilitation involve a trade-off between functional benefits, emotional comfort, and perceived risks.” Exercise rehabilitation preferences, therefore, play both motivating and regulatory roles.

I prefer using rehabilitation equipment for recovery. With the help of equipment, I can exercise more effectively and persist more easily. (P1)

At first, I bought elastic bands for exercise, but I felt weak and worried that my movements might not be accurate and could hurt me. I prefer low-intensity exercises like walking and finger-grip training, which my body can adapt to more easily, unlike high-intensity training, which can make people feel resistant. (P6)

I enjoy going out to exercise. After getting sick, I do not have much motivation to exercise alone at home. Going out allows me to chat with others, supervise and encourage each other, and makes exercise more enjoyable. (P8)

I like following rehabilitation videos when exercising. I have never been exposed to this kind of knowledge before, so following the videos makes me feel more at ease. It also helps prevent injuries caused by incorrect movements when exercising alone. (P17)

#### 3.1.5 Economic pressure burden

Patients with stroke-related motor dysfunction often face a prolonged rehabilitation process. Their economic expenditure on home-based exercise rehabilitation exhibits a dual structure of explicit and implicit costs. This financial burden may directly compel patients to make passive compromises in accessing rehabilitation resources and implementing rehabilitation plans. Over time, such compromises can trigger feelings of guilt and self-denial, ultimately hindering rehabilitation progress.

My daughter wanted to buy me a fingerboard that costs just over a hundred yuan, but I did not let her buy it. I told her, ‘It is useless—practicing would be in vain.’ In fact, I just felt bad about spending the money. (P8)

Sometimes, when I cannot sleep at night, I look at my weak hands and feel like a burden. If it were not for me, life at home would be easier. Now, my desire to recover is weakening. Let it be. (P9)

Professional rehabilitation equipment is not covered by medical insurance, and buying it on my own is too expensive. So I can only afford basic equipment like elastic bands, but I worry that I will not be able to control the force properly. If my financial situation were to improve, I would not hesitate. (P13)

I told my wife, ‘I am not going to practice anymore. We can save some money and buy toys for our grandson.’ I feel anxious inside, but when I think about the cost, I lose the motivation to keep going. My legs still are not steady enough to walk, and I feel like this might be the end of my life. (P7)

### 3.2 Driving and constraints of behavior-related cognition

#### 3.2.1 Perceived effects of rehabilitation effects

Perceived rehabilitation effects refer to stroke survivors’ subjective judgments and cognitive evaluations during home-based exercise rehabilitation, based on changes in physical function, daily activity abilities, and subjective feelings after training. These perceptions directly influence motivation for rehabilitation and behavioral persistence.

Positive perceptions of rehabilitation effects.

Now I can slowly drink water from a cup by myself. Although my movements are not yet very flexible, I can genuinely feel the improvement. (P2)

The rehabilitation has been effective. At first, I could not perform fine movements, such as holding a cup of water. Now I can basically do them independently, which gives me hope. I now arrange my own exercises every day without needing my family to remind me. (P6)

Negative perceptions of rehabilitation effects.

My physical condition is like this now. At the beginning, I noticed some improvement, but later, no matter how much I trained, there was no further progress. Gradually, I lost interest in continuing to exercise. (P4)

I had hoped to walk independently sooner, but now it seems like there has been no progress at all. I do not think these exercises are helpful, and I never feel motivated when practicing. Sometimes I want to stop halfway, feeling like there is no end to this recovery journey. (P12)

#### 3.2.2 Rehabilitation self-efficacy

In home-based exercise rehabilitation for stroke patients, rehabilitation self-efficacy primarily manifests as patients’ perceived controllability over their rehabilitation process.

Some patients attempted to break down their rehabilitation goals into smaller, achievable micro-goals. Accomplishing these micro-goals fostered higher rehabilitation self-efficacy, enabling patients to better cope with obstacles during home-based exercise rehabilitation.

Now I need a walker to walk, and I always feel tired when lifting my leg. However, every time I take even half a step more, I feel like I have made progress again. These small improvements give me confidence in my recovery and make me more willing to stick to the plan. (P1)

At first, I could not walk steadily and fell once while practicing balance. However, I did not give up. I broke my goal into smaller steps and practiced step by step. I started by walking a few steps while holding onto the wall, gradually increasing the distance. Each small success made me feel I was progressing and could control the pace myself, which boosted my confidence. (P11)

Conversely, some patients compared their current functional impairments with their pre-stroke condition and attempted activities beyond their physical capacity. This often led to frustration, self-doubt, and even discontinuation of rehabilitation activities.

After getting sick, I wanted to recover quickly and return to how I was before, so I tried walking with support. However, I was not steady and fell. Now I am particularly afraid of falling, so I do not dare exercise much and gradually exercise my lower limbs less. (P4)

In the past, lifting my arms and wiping the table were effortless. Now, when I try to lift my arms, my hands shake much more than before… It feels like my body is just like this, so I might as well save some energy. (P5)

#### 3.2.3 Adaptability of the rehabilitation environment

Environmental adaptability refers to the degree of alignment between ecological factors—such as physical space and interpersonal support—and the rehabilitation needs of stroke survivors in home-based exercise programs. This adaptability directly affects the convenience, safety, and sustainability of rehabilitation and is a critical external condition for the smooth continuation of rehabilitation behaviors.

A space in my living room has been specially set aside for me to practice. Now it is very convenient to do rehabilitation exercises every day. I do not need to look for a place, and I am not afraid of falling, so I can practice with peace of mind. (P10)

My son helped me print the training plan in large font and posted it on the refrigerator. He also saved a few simple rehabilitation videos on my phone, so whenever I do not know what to practice, I can refer to them. (P1)

However, inadequate adaptability of the rehabilitation environment can constrain patients’ initiative, leading to reduced engagement or even the interruption of rehabilitation activities.

My house is small, so there is no space for me to practice walking. I can only shuffle a few steps in the narrow hallway and have to be careful not to knock things over. (P15)

Without professional support in the community, we can only rely on ordinary fitness equipment. However, this equipment is not designed for stroke patients, and being afraid of making incorrect movements that could worsen my condition makes me hesitant. Over time, this fear makes me lazy and unwilling to move. (P7)

#### 3.2.4 Multidimensional information asymmetry

Multidimensional information asymmetry represents a core dilemma faced by stroke patients during home-based exercise rehabilitation. It encompasses several dimensions—such as information on rehabilitation needs, available resources, service quality, and welfare policies—that collectively hinder effective access to and utilization of rehabilitation services. This asymmetry weakens patients’ initiative and adherence with rehabilitation behaviors.

When I was discharged from the hospital, the doctor told me to ‘go home and practice more physical exercises.’ However, I had no idea which part of my body I should focus on—my arms or my legs—and I did not know what level of practice was appropriate. (P9)

I have difficulty walking, so going out to rehabilitation institutions is very troublesome for me. It would be helpful if rehabilitation therapists could provide regular on-site guidance, but I do not know how to contact them. As a result, I can only practice at home, and I am afraid to try many exercises, fearing I might do something wrong. (P4)

There are many rehabilitation methods available online now, but I cannot tell which ones are scientific and which might be misleading. I am worried about injuring myself by doing the wrong exercises, so I only dare to perform a few simple exercises each time. I feel that it is hard to achieve any real effect this way. (P15)

My grandson mentioned that there is a policy for “renting rehabilitation aids for people with disabilities,” such as walkers and training chairs, which could save a lot of money. However, when I asked at the community neighborhood committee, they were not clear about it either. (P13)

### 3.3 Moderating effect of behavior-related emotional responses

#### 3.3.1 Rehabilitation of emotional experience

Rehabilitation emotional experience refers to a range of subjective emotional responses and psychological states experienced by stroke patients, shaped by their rehabilitation progress, interactions with the environment, self-perceptions, and related experiences. These emotional responses constitute an important psychological driving force in the home-based exercise rehabilitation process.

Positive emotional experiences often provide patients with a sense of accomplishment and joy, thereby promoting sustained engagement in rehabilitation behaviors.

At first, my hands would not obey me at all. I never thought I would be able to button my clothes slowly by myself one day. That moment gave me a great sense of accomplishment and motivated me to keep exercising. I was able to complete my rehabilitation plan every day. (P2)

In the beginning, my legs were very weak, but later I was able to walk slowly on my own. I even wrote it down that day because I felt extremely happy. Now I do not need my family to remind me to exercise on time. (P1)

In contrast, negative emotional experiences can trap patients in feelings of frustration and helplessness, becoming significant barriers to rehabilitation behaviors.

Friends who got sick around the same time can now go out for walks, but I still need crutches. The more I practice, the more useless I feel. I cannot keep up with them, so I gradually become lazy and stop exercising. (P4)

I used to enjoy chatting and exercising with my neighbors downstairs, but now I need crutches and find it difficult to climb stairs. I had hoped for a quick recovery, but I cannot even manage simple daily tasks. Now that I stay at home, I do not feel motivated to exercise. (P12)

#### 3.3.2 Family support

Family support is a critical factor influencing patients’ confidence in their rehabilitation and their participation in training. Most participants reported that encouragement, supervision, reminders, and emotional support from family members enhanced their confidence and motivated them to engage actively in rehabilitation.

After suffering a cerebral infarction, I did not want to go out or interact with others. However, my husband accompanies me every day, encourages me, and supervises me. With him by my side, I am willing to go for a walk and persist in exercising. (P3)

My family never urges me to ‘get better quickly’ or puts pressure on me. Instead, they always encourage and supervise me. Without their support, I would not have been able to persist or recover so well. (P8)

However, effective family support does not equate to excessive involvement. Some participants reported that overprotection or excessive interference by family members diminished their sense of autonomy in rehabilitation.

When I go out for a walk, I want to take it slow and steady, but it keeps urging me to ‘walk faster’ and will not listen even when I explain. I used to ask him to accompany me, but now I feel annoyed whenever it comes to walking. (P13)

My shoulder hurts badly now, but my family believes that more practice will help and keeps urging me to exercise. In fact, the more I practice, the worse it feels, and now I have developed a strong aversion to exercising. (P14)

#### 3.3.3 Peer support

Peer support facilitates emotional connection and trust through shared experiences and similar social backgrounds.

Several participants indicated that positive peer support helped alleviate feelings of loneliness, strengthened confidence in recovery, and enhanced adherence to exercise rehabilitation.

In our community clinic, the acupuncture and massage areas are mostly occupied by stroke patients. We often communicate and encourage each other. Recovering alongside others makes us feel less alone and gives us more motivation to keep going. (P2)

I have a friend who also had a stroke. Because he was lazy and did not exercise properly, he now needs someone to care for him. Whenever I feel like slacking off, I think of him and immediately get up and move. I do not want to follow his path. (P17)

Conversely, some patients felt that peer support was insufficient or that advice from fellow patients lacked professional credibility, which could negatively affect rehabilitation behaviors.

*When I was hospitalized, I exercised and encouraged other patients. However, after returning home, there was no one to talk to. Practicing alone felt boring, and sometimes I forgot to exercise, which gradually made me less serious about it. (P5)*

*Other patients with cerebral infarction just say, ‘Go out and move around more,’ but I did not take it seriously. My exercise was irregular, and now I can only walk with a cane. (P12)*

## 4 Discussion

This study explored the complex processes experienced by stroke patients during home-based exercise rehabilitation, examined their rehabilitation behaviors, and identified three major themes: the multidimensional influence of personal characteristics and past experiences; the driving and constraints of behavior-related cognition; and the moderating effect of behavior-related emotional responses. These findings are consistent with those of previous studies (25–28). However, the present study specifically focuses on the mechanisms underlying behavioral transformation in home-based exercise rehabilitation and, guided by the HPM, provides a novel interpretive framework and intervention perspective for understanding this complex process.

Our findings indicate that physical function, family roles, and economic status—particularly self-decision-making ability and exercise rehabilitation preferences—jointly shape initial motivation and basic patterns of exercise rehabilitation behavior. Notably, limited self-decision-making ability emerged as a key factor restricting active participation in rehabilitation. Patients often experience decision-making dilemmas due to insufficient information and cognitive biases, leading to interruptions in or the discontinuation of rehabilitation, a finding consistent with that of Roaldsen et al. (29). At present, discharge guidance in clinical practice is largely standardized propaganda and education, often neglecting the cultivation of the decision-making ability of patients. As a result, patients may struggle to independently address rehabilitation-related challenges, thereby reducing their willingness to engage in recovery actively (30). Healthcare professionals can leverage digital platforms and employ decision-making aids—such as pros-and-cons analysis forms and question prompt lists—to enhance the decision-making awareness of patients and facilitate a transition from passive acceptance to active participation. Importantly, the long-term coexistence of economic pressures among stroke patients significantly affects the process of home-based exercise rehabilitation, a finding consistent with that of Cheng et al. (31). Our study reveals that economic stress is not merely an objective financial issue but also a complex psychosocial challenge intertwined with family responsibilities and perceptions of self-worth. Future efforts should explore low-cost support models, strengthen links with community resources, and leverage government subsidies or charitable funding to reduce decision-making burdens and improve access to rehabilitation services. In addition, home-based exercise rehabilitation behaviors are influenced by both subjective and objective factors, including personal habits and disease status. Patients often exhibit strong preferences regarding exercise modes, environments, and assistive tools. Research shows that personalized exercise rehabilitation programs that account for the preferences of patients can enhance positive experiences and improve adherence (32). However, in practice, traditional rehabilitation services often rely on standardized processes, overlooking individual needs (33). This creates a pronounced mismatch between the “heterogeneity” of patients’ functional impairments and the “homogeneity” of rehabilitation interventions, resulting in poor adherence and suboptimal outcomes in home-based rehabilitation. Therefore, rehabilitation strategies should shift from a “standardized” approach to a “personalized,” patient-centered model that respects individual preferences and decision-making needs, thereby fostering positive rehabilitation experiences and promoting the initiation and maintenance of active exercise rehabilitation behaviors.

Furthermore, our analysis demonstrates that the patients’ perceptions of rehabilitation effectiveness, self-efficacy, adaptability of the rehabilitation environment, and information asymmetry directly influence their confidence, effort, and sustainability in performing rehabilitation tasks. Cognitive confusion and uncertainty were identified as core contributors to behavioral discontinuity. Home-based exercise rehabilitation following stroke is a longitudinal and multifaceted process involving multiple components and stakeholders, including goal setting, functional assessment, formulation and implementation, use of assistive devices, and supervision with feedback (34). This study found that when patients clearly perceive the direct benefits of rehabilitation for functional recovery, their intrinsic motivation is significantly enhanced, leading to greater participation and adherence—consistent with previous findings (35). Consequently, healthcare providers should prioritize patients’ subjective rehabilitation experiences, guide them in recognizing exercise-related benefits, provide timely feedback and evaluation, and enhance rehabilitation self-efficacy. It is also noteworthy that inadequate adaptation of the rehabilitation environment and multidimensional information asymmetry are major barriers limiting access to home-based rehabilitation services. During home-based rehabilitation, stroke patients are often exposed to unstable external conditions that can undermine adherence, delay functional recovery, or even lead to functional decline. From an environmental and resource perspective, insufficient adaptation of the rehabilitation setting reduces service accessibility. It contributes to the marginalization of patients and their families after discharge, thereby constraining effective home-based rehabilitation (36). Previous studies have shown that stroke patients have substantial unmet needs for rehabilitation-related information, with limited access to information after discharge and insufficient knowledge and skills related to stroke rehabilitation (37). Our findings further indicate that patients face broader information gaps beyond basic knowledge deficits, including multidimensional asymmetries in local rehabilitation pathways, community support policies, and welfare application procedures. In the current context, rehabilitation information systems are fragmented, dispersed, and insufficiently targeted, making it difficult for patients to obtain relevant information and posing a significant barrier to home-based self-rehabilitation (38). With advances in information technology, mobile health and telemedicine have increasingly emerged as key drivers of home-based healthcare for patients with chronic diseases and may offer viable solutions to existing gaps in rehabilitation services (39, 40). As demonstrated in this study, optimizing the information environment and feedback mechanisms and fostering a clear and supportive cognitive context may help address current behavioral and cognitive challenges. Accordingly, it is recommended to expand access to rehabilitation information, strengthen comprehensive, full-cycle stroke rehabilitation support systems, and fully leverage digital health strategies—such as mobile health applications and telemedicine—to provide continuous support, including remote guidance, dynamic monitoring, information dissemination, and resource linkage. These measures may effectively bridge rehabilitation information gaps and better meet the diverse needs of patients undergoing home-based exercise rehabilitation.

At the same time, our findings indicate that emotional experiences during home-based exercise rehabilitation, as well as emotional support from family members and peers, are important regulatory factors influencing the maintenance or withdrawal of rehabilitation behaviors. Interview analysis revealed that the emotional experiences of stroke patients during home-based exercise rehabilitation are complex and diverse. Patients not only experience negative emotions such as anxiety and depression due to disease and other factors but also develop positive emotions, including a sense of achievement and pleasure, through rehabilitation. As a significant and decisive driving force, emotions profoundly influence individual cognition and behavior (41). Zhang et al. (42) reported that positive psychological capital can provide essential psychological resources for the formation of positive cognition following trauma, thereby enhancing the rehabilitation experiences and initiative of patients. Similarly, Zhong et al. (43) demonstrated that cognitive behavioral therapy can effectively alleviate negative emotions such as anxiety and depression by guiding patients to identify and correct irrational cognitions related to disease and rehabilitation, thus improving emotional regulation and psychological adaptability. These findings suggest that healthcare professionals should prioritize the emotional experiences of patients by providing timely psychological counseling and emotional support. Through cognitive behavioral therapy and positive psychological interventions, clinicians can help patients manage negative emotions, activate internal psychological resources, and ultimately improve participation and adherence to home-based exercise rehabilitation. Moreover, social and emotional support from multiple sources is a key determinant of sustained rehabilitation behavior. Calderon et al. (44) noted that effective external emotional support can enhance the well-being and perceived benefits of patients, motivating them to adopt proactive coping strategies. As primary social units with strong emotional bonds, families provide both physical and emotional support that is essential for strengthening the rehabilitation resilience of patients and positive coping capacities (45). In addition, peer emotional communication plays a unique role in alleviating negative emotions and improving rehabilitation adherence (46). Studies have shown that online peer support groups can effectively mitigate emotional challenges and promote long-term adherence to rehabilitation by fostering emotional resonance, narrative hope, and empathic interaction, thereby transforming emotional distress into sustained social support (47). These findings indicate that healthcare professionals should actively integrate multiple external resources, strengthen communication and education for family members, and encourage their involvement in rehabilitation decision-making to establish a multidisciplinary, family-centered collaborative support model. Simultaneously, social media groups or online rehabilitation support communities can be developed to facilitate peer communication and information sharing, thereby enhancing external motivation and promoting the transformation and maintenance of rehabilitation behaviors.

To the best of our knowledge, this is the first qualitative study to apply the Pender’s HPM to explore the factors influencing home-based exercise rehabilitation behaviors among stroke patients, thereby expanding the application of this theoretical framework to specific rehabilitation contexts. Our findings confirm the applicability of the HPM for understanding complex health behaviors, as its framework effectively captures multilevel influences ranging from the internal characteristics of individuals to the external social environments. These findings may inform the development of feasible, targeted behavior-change interventions to promote active rehabilitation among stroke patients in home settings. However, our findings also suggest that, in chronic disease management contexts such as home-based rehabilitation—which are deeply embedded in social systems—the theoretical model may need to place greater weight on structural factors, including socioeconomic constraints and macro-level information barriers. Although such factors can be partially categorized as “situational influences,” their pervasive and restrictive impact warrants greater emphasis in theoretical development. Future research should explore more systematic approaches to integrating these structural dimensions into health behavior theories to enhance their explanatory power and practical relevance for real-world public health challenges.

### 4.1 Limitations

This study has several limitations that should be acknowledged. First, all participants were recruited from the same hospital. Although maximum variation sampling was employed, the findings may not fully capture the diversity of stroke patients, potentially limiting the generalizability of the results. Future studies should adopt multicenter designs and include more diverse patient populations. Second, qualitative research inherently involves subjectivity, and the personal experience, cognitive abilities, and analytical approaches may influence findings. In future research, mixed-methods designs that combine qualitative and quantitative approaches may help mitigate this limitation. The integration of quantitative data can complement qualitative findings, enhance methodological rigor, and improve the robustness of conclusions. Finally, as highlighted in this discussion, further investigation into the mechanisms underlying behavioral transformation in home-based exercise rehabilitation among stroke patients represents a critical and promising direction for future research.

## 5 Conclusion

This study conducted semi-structured interviews with 17 stroke survivors, guided by the health promotion model, to explore the factors influencing home-based exercise rehabilitation behavior. Building on these findings, the study further examined the identified multilevel influencing factors and proposed targeted coping strategies. These strategies provide both a theoretical and practical foundation for developing comprehensive intervention strategies to promote active rehabilitation behaviors and improve home-based exercise rehabilitation.

## Data Availability

The original contributions presented in the study are included in the article/supplementary material, further inquiries can be directed to the corresponding author.
